# Revealing a Phenotypical Appearance of Ibrutinib Resistance in Patients With Chronic Lymphocytic Leukaemia by Flow Cytometry

**DOI:** 10.3389/pore.2022.1610659

**Published:** 2022-09-21

**Authors:** Ferenc Takács, Lili Kotmayer, Ágnes Czeti, Gábor Szalóki, Tamás László, Gábor Mikala, Ágnes Márk, András Masszi, Péter Farkas, Márk Plander, Júlia Weisinger, Judit Demeter, Sándor Fekete, László Szerafin, Beáta Margit Deák, Erika Szaleczky, Adrienn Sulák, Zita Borbényi, Gábor Barna

**Affiliations:** ^1^ Department of Pathology and Experimental Cancer Research, HCEMM-SE Molecular Oncohematology Research Group, Semmelweis University, Budapest, Hungary; ^2^ Center for Pathology, University Medical Center—University of Freiburg, Freiburg, Germany; ^3^ South-Pest Central Hospital—National Institute for Hematology and Infectious Diseases, Budapest, Hungary; ^4^ Department of Internal Medicine and Hematology, Semmelweis University, Budapest, Hungary; ^5^ Department of Hematology, Markusovszky University Teaching Hospital, Szombathely, Hungary; ^6^ Department of Internal Medicine and Oncology, Semmelweis University, Budapest, Hungary; ^7^ Hospitals of Szabolcs-Szatmár-Bereg County and University Teaching Hospital, Nyíregyháza, Hungary; ^8^ National Institute of Oncology, Budapest, Hungary; ^9^ 2nd Department of Internal Medicine and Cardiology Center, University of Szeged, Szeged, Hungary

**Keywords:** flow cytometry, targeted therapy, drug resistance, chronic lymphocytic leukaemia, ibrutinib

## Abstract

**Background:** Ibrutinib is widely known as an effective and well-tolerated therapeutical choice of the chronic lymphocytic leukaemia (CLL). However, acquired resistance may occur during the treatment, causing relapse. Early detection of ibrutinib resistance is an important issue, therefore we aimed to find phenotypic markers on CLL cells the expression of which may correlate with the appearance of ibrutinib resistance.

**Methods:** We examined 28 patients’ peripheral blood (PB) samples (treatment naïve, ibrutinib sensitive, clinically ibrutinib resistant). The surface markers’ expression (CD27, CD69, CD86, CD184, CD185) were measured by flow cytometry. Furthermore, the BTK^C481S^ resistance mutation was assessed by digital droplet PCR. Moreover, the CLL cells’ phenotype of a patient with acquired ibrutinib resistance was observed during the ibrutinib treatment.

**Results:** The expression of CD27 (*p* = 0.030) and CD86 (*p* = 0.031) became higher in the clinically resistant cohort than in the ibrutinib sensitive cohort. Besides, we found that high CD86 and CD27 expressions were accompanied by BTK^C481S^ mutation. Our prospective study showed that the increase of the expression of CD27, CD69 and CD86 was noticed ahead of the clinical resistance with 3 months.

**Conclusion:** Our study suggests that the changes of the expression of these markers could indicate ibrutinib resistance and the examination of these phenotypic changes may become a part of the patients’ follow-up in the future.

## Introduction

Chronic lymphocytic leukaemia (CLL) is the most common leukaemia in the Western world (1, 2). Although CLL has remained an incurable disease, with the appearance of new innovative drugs such as ibrutinib (IBR) as an irreversible Bruton’s tyrosine kinase (BTK) inhibitor the therapeutic landscape has changed (3). While chemo-immunotherapy appeared to be ineffective among patients with harbouring del17p or TP53 mutation, IBR has shown remarkable efficiency in this patient cohort as well (4, 5). IBR monotherapy is a highly effective way to treat CLL, but continuous treatment is required to maintain the remission, raising the possibility of evolving drug resistance (6). This is a frequent and troublesome problem, that is why the mechanisms of the resistance have become an appealing target of investigation in the recent years. A point mutation of the *BTK* gene, as the most frequent C481S missense mutation, could inhibit IBR to bind to the BTK, resulting in treatment failure. Examining this mutation is a well-known approach to predict resistance (7,8,9). Besides the molecular techniques, flow cytometry seems to be a promising approach to monitoring the treatment response. The detection of measurable residual disease (MRD) by flow cytometry has been remarkably effective, predicting the treatment response in case of venetoclax (VEN) monotherapy. However, undetectable MRD level has been achieved considerably less often in case of ibrutinib treatment (10,11,12). Therefore, there is still a need for finding markers that can not only be easily measured by flow cytometry but can also predict IBR resistance. Tissino et al. pointed out that CD49d could be a promising predictive factor of ibrutinib treatment. According to their data, patients had shorter progression free survival when CLL cells in peripheral blood were CD49d positive (13). According to literature, beyond CD49d there are numerous cell surface markers (i.e. CD69, CD184, CD185, CD27, and CD86) which may have prognostic or predictive relevance in CLL. CD69 has shown increased expression on CLL cells, and this feature has been accompanied with increased CD38 expression (14). In addition, it has been shown in an *in vivo* cell culture study that CD69 expression decreased due to ibrutinib treatment (15). Other *in vivo* studies have shown that the CD184 and CD185 could be involved in the B-cell receptor (BCR) signalling. On the one hand, CD184 expression decreased due to ibrutinib (16), while CD185 enhanced the BCR-triggered B-cell activation (17). As far as CD27 is concerned, its expression on CLL cells was increased by ibrutinib in an *in vitro* cell culture study (18). Finally but importantly, CD86 has been considered a potentially novel prognostic factor of CLL (19, 20). These markers seem to be promising prognostic or predictive factors; however, they have been poorly investigated in the real-world cohort of patients with CLL under ibrutinib monotherapy. For this reason, we addressed the question whether the expression level of CD69, CD184, CD185, CD27 and CD86 is different among treatment naïve, ibrutinib sensitive and ibrutinib-resistant cohorts of patients or not. Moreover, we wanted to know if there is any connection between the expression of these markers and *BTK* ^C481S^ resistance mutation in case of clinical ibrutinib resistance.

## Materials and Methods

### Patients and Samples

Peripheral blood samples were collected from treatment naïve (Co) (*n* = 10, female/male ratio 3/7; median age 69 years (55–83)), ibrutinib sensitive (IS) (*n* = 7 female/male ratio 5/2; median age 72 years (63–86)) and clinically ibrutinib-resistant (IR) (*n* = 11 female/male ratio 2/9; median age 70 years (56–87)) CLL patients in six Hungarian oncohaematological centers. The investigational period took from 01.09.2018 to 31.08.2021 and the diagnosis of CLL was based on the current WHO guideline (21). During this investigational period 10 treatment naïve CLL patients were selected as control patients at various times. All ibrutinib treated patients received the drug in a daily dose of 420 mg as a single-agent therapy. The patients of IS cohort were treated with IBR exactly for 1 year, and the samples were taken after this 1-year IBR treatment. The samples were negative for *BTK* ^*C481S*^ mutation at that time. Patients were followed in accordance with the institutional protocols of the participating institutions. Commonly follow up visits were performed every 3 months. Clinical response to treatment was determined according to the iwCLL guideline (22). Complete or partial remission was accepted as clinically significant response. Loss of the best response achieved was defined as relapse hence resistance to Ibrutinib. The IR group patients were treated with ibrutinib for a minimum 4 months (median 28.5 months, range: 4–57 months), and the samples were taken when the patients were considered as ibrutinib resistant. Clinical characteristics are summarised in Supplementary Tables S1–S3 In an earlier case-study, we pointed out that the increase of CD86 expression on CLL cells could predict the VEN resistance prior to the onset of the appearing of the clinical resistance (23). For this reason, we were wondering whether CD86 might show a similar feature during ibrutinib treatment, and we decided to carry on the follow-up of this patient during the IBR monotherapy. In this case, ibrutinib was started as a salvage therapy with a 420 mg daily dose due to venetoclax resistance (day 0). We measured the expression of CD27, CD69, and CD86 on days 0, 30, 90, 120, 240, 330 and on the onset of the clinical resistance (day 420). The clinical characteristics of the follow-up patient are seen in Supplementary Table S4. This patient has not been enrolled in the IR cohort because VEN treatment could eradicate the *BTK* ^C481S^ mutated clones (8, 9), and enrolling patients with prior VEN treatment could strongly confuse our current molecular study.

### Compliance With Ethical Standards

The patients were informed in writing, and written consent was obtained from all participants. The study was approved by the Hungarian Medical Research Council (ID:45371-2/2016/EKU) and it was conducted in accordance with the Declaration of Helsinki.

### Flow Cytometry

We used the stain-lyse-wash procedure to prepare the PB samples for flow cytometric acquisition. 100-μl PB samples were incubated with the antibodies against surface epitopes for 13 minutes. The antibodies used in our experiments are listed in the (Supplementary Table S5) Then the samples were lysed by BD FACS^TM^ Lysing Solution (Beckton Dickinson Biosciences (BD) CA USA) for 15 minutes. Before the acquisition, all samples were washed twice (5 min, 400 g, room temperature) by phosphate-buffered saline (PBS, 137 mM NaCl, 2.7 mM KCl, 10 mM Na2HPO4, 1.8 mM KH2PO4, pH = 7.4). All used antibodies were pre-titrated, fluorescent staining was carried out in the dark, and stained samples were protected from light. Samples were measured by using an 8-color Navios flow cytometer (Beckman Coulter (BC) FL, USA). Instrument settings were regularly controlled by Flow-Set Pro and Flow-Check Pro QC beads (BC). To obtain a satisfactory number of CLL cells, at least 50,000 total events were acquired in each sample. Flow data were analysed using Kaluza 2.1.1 software (BC). Living cells, lymphocytes, monocytes, and granulocytes were identified based on side scatter (SSC) and CD45 dot-plot. Finally, the proportion of CLL cells among CD19 positive lymphocytes was assessed based on CD5 expression. The CLL cell ratio among B-cells was above 98% in each sample, therefore CLL cells were considered as B-cells (Supplementary Figure S1).

We could not perform measurements with isotype controls from every sample due to a high number of investigated markers and low available volume of PB samples, but the feasibility and specificity of antibodies were tested. Therefore, we had to establish a novel gating strategy using internal controls to calculate the median fluorescence intensity value (MFI). This novel approach to measure MFI was based on our previously published work (23). According to our measurements, CD19 negative lymphocyte population was suitable as an internal negative control for CD69, CD184, CD185, and CD86. Concerning CD27, the granulocyte population was used as internal negative control. Relative MFI values were calculated as the difference between the MFI value of internal negative controls and B-cells (Supplementary Figure S2). These differences between the MFI values are displayed on the graphs.

### Detection of the BTK^C481S^ Resistance Mutation

*BTK* ^C481S^ resistance mutation was previously determined from the samples by digital droplet PCR (ddPCR; Bio-Rad Laboratories, CA, USA) (8, 9, 24). In each sample, 100 ng input DNA was used and all reactions were carried out according to the manufacturer’s instructions. Droplets were generated by the QX200 Automated Droplet Generator followed by fluorescent signal detection using the QX200 Droplet Reader system. Results were evaluated and quantified using the Bio-Rad QuantaSoft software. The cut-off variant allele frequency (VAF) level was 0%. Samples were considered positive for *BTK* ^C481S^ if the mutation was detected with a VAF higher than the 0% cut-off (Supplementary Figure S3). *BTK* mutation status of all patients from the IR group was published previously by our research group (24).

### Statistical Analysis

We used SigmaPlot 12.5 (Systat Software Inc. CA, USA) for graphing and statistical analyses. Normality (Shapiro-Wilk) and equal variance tests were performed in each case, followed by ANOVA, Kruskal-Wallis-tests with Holm-Sidak post hoc test, t-test or Mann-Whitney-test based on their results. Differences were considered statistically significant at *p* < 0.05.

## Results

### Ibrutinib’s Effect to the Immunophenotype of CLL Cells

**FIGURE 1:**
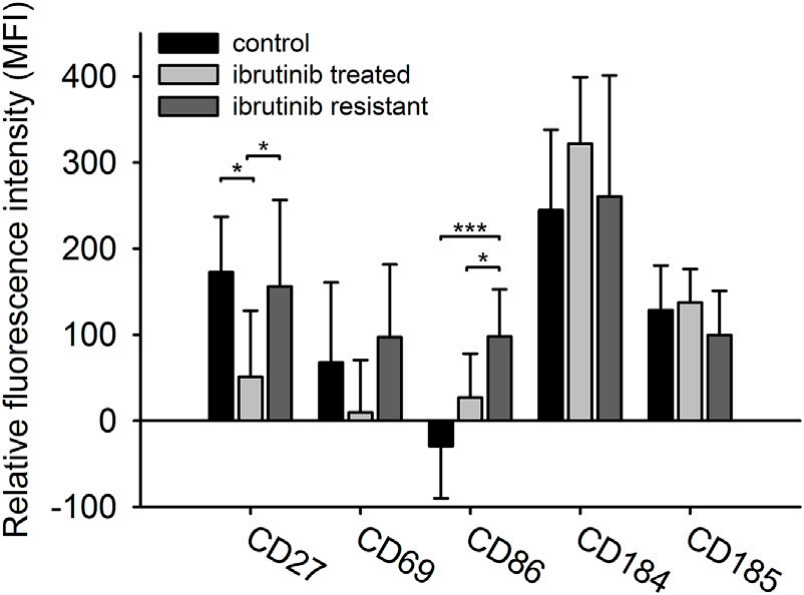
The immunophenotype of CLL cells in different treatment cohorts. The expression level of five different surface markers (CD69, CD184, CD27, CD86, CD185) in three different cohorts (treatment naïve *n* = 10, ibrutinib sensitive *n* = 7, ibrutinib resistant *n* = 11) were measured by flow cytometry. Relative median fluorescent intensity (MFI) values were calculated as the difference between the MFI value of internal negative controls and B-cells. ANOVA or Kruskal-Wallis test with Holm-Sidak post hoc test were performed for statistical evaluation. *p < 0.05; **p < 0.01.

In our study we compared the immunophenotypes of treatment naïve (Co), ibrutinib sensitive (IS), and clinically ibrutinib-resistant (IR) cohorts of CLL patients. We investigated five surface markers’ expression (CD27, CD69, CD86, CD184, CD185) in these cohorts. Although only CD27 and CD86 have shown significant differences in some cohorts, but CD69 expression’s change seemed also to be interesting (Figure 1). The expression of CD27 was significantly lower in the IS group compared to both the Co group (IS vs. Co: 51.136 vs. 172.709 MFI values, *p* = 0.020) and the IR group (IS vs. IR: 51.136 vs. 156.341 MFI values, *p* = 0.030). In case of CD69 expression, although we did not find significant differences between the IS and IR group, but CD69 has shown remarkably higher expression in the IR group compared to the IS group (IR vs. IS: 97.238 vs. 9.919 MFI values, *p* = 0.111). Comparing the expression levels of CD86 between the IS and Co groups (IS vs. Co 27.23 vs. −29.308 MFI values, *p* = 0.052), the detected values tended to be significant. Additionally, CD86 expression was significantly higher in IR group compared to IS group (IR vs. IS 97.788 vs. 27.23 MFI values, *p* = 0.031).

**FIGURE 2:**
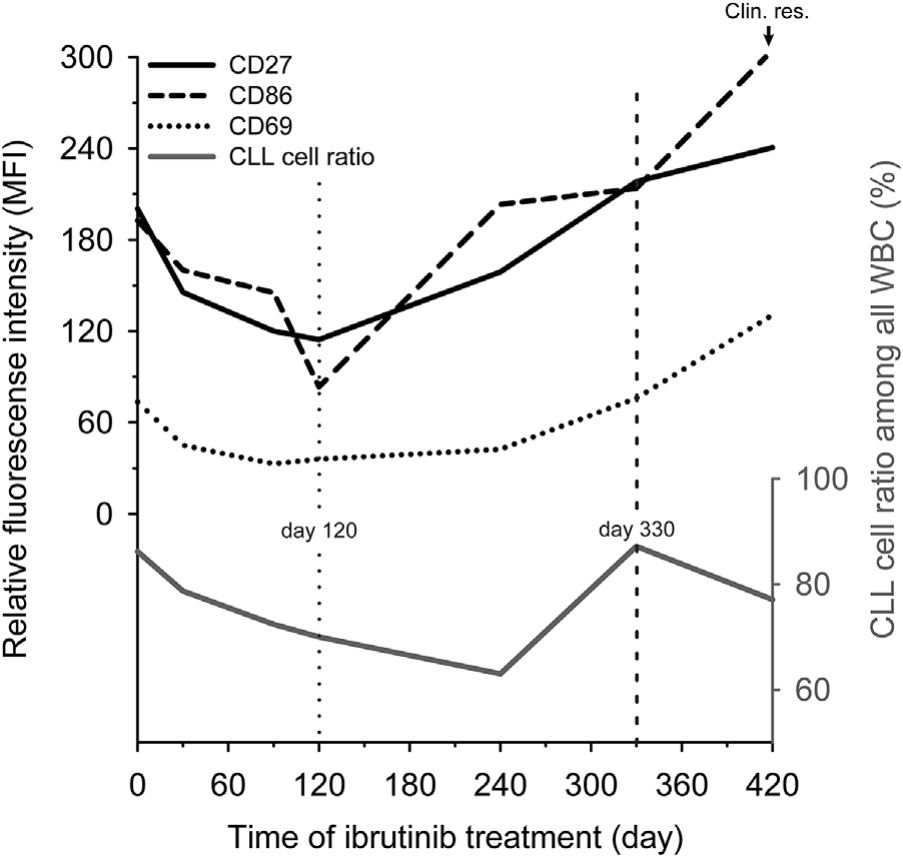
The change of CD27, CD69 and CD86 expression during ibrutinib treatment. Relative expression levels of three markers (CD27 (black line), CD69 (dotted line) and CD86 (dashed line)) and the CLL ratio (grey line) were analysed during ibrutinib treatment in a patient’s peripheral blood sample who ultimately became ibrutinib resistant (Clin. res.). We used the initial expression levels (day 0) of the CD27, CD69 and CD86 as a benchmark. The time point when the tendency has changed level marked by a vertical dotted line. When the markers’ expression exceeded the benchmark level marked by a vertical dashed line.

We observed the changing of the expression pattern of the previously mentioned markers during ibrutinib treatment in a follow-up patient. We used the initial expression level (day 0) of the CD27, CD69 and CD86 as a benchmark. In the beginning Ibrutinib monotherapy seemed to be effective and the expression of all the above-mentioned markers was continuously decreased, reaching their lowest level by day 120. Since then, the expression of these markers tendentially increased, exceeding the benchmark by day 330 (MFI values on day 330 vs. day 0: CD27: 218.26 vs. 200.42, CD69: 75.86 vs. 73.46, CD86: 213.85 vs. 192.56). The sign of clinical resistance, i.e. enlarged lymph nodes appeared by day 420 and the expression of CD27, CD69 and CD86 also reached their maximum level on day 420 (MFI values on day 420; CD27: 240.57, CD69: 130.58, CD86: 304.41). We also observed that the CLL cell ratio changed independently of the markers’ expression level during the ibrutinib treatment (Figure 2).

### Elevated CD27 and CD86 Expression in BTK^C481S^ Mutated CLL Samples

**FIGURE 3:**
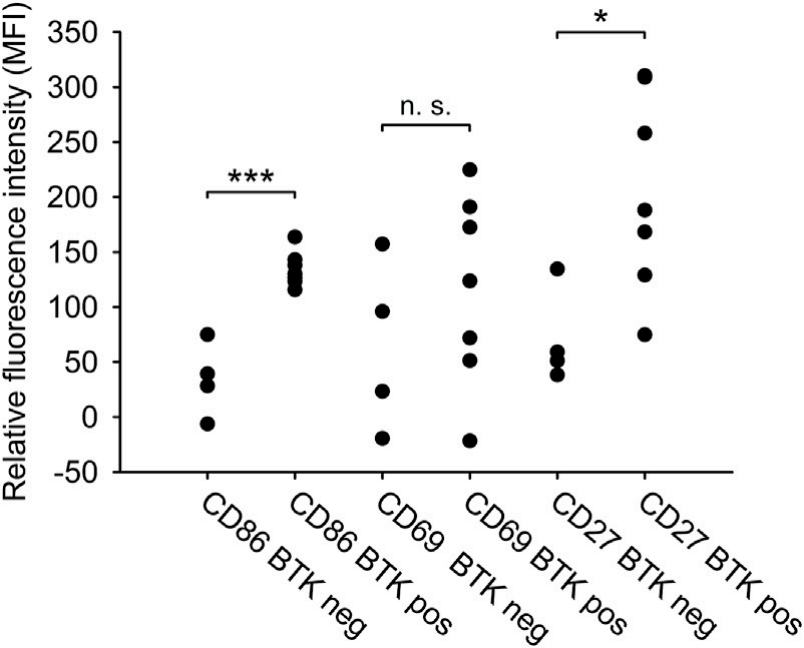
Phenotypic difference between *BTK* ^*C481S*^ *mutated and wild type CLL samples*. The relative expression of CD27, CD69 and CD86 surface markers and the *BTK* ^C481S^ mutation status in the clinically ibrutinib-resistant patients (*n* = 11) were compared. T-test or Mann-Whitney-test were performed for statistical evaluation. *p < 0.05; **p < 0.01.

We compared the *BTK* ^*C481S*^ mutation status with the expression of the three selected markers (CD27, CD69, CD86) of clinically ibrutinib resistant patients in order to reveal a potential connection between the *BTK* ^*C481S*^ mutation status and the immunophenotype of CLL cells. 64% of ibrutinib resistant cases (7/11) harboured the *BTK* ^C481S^ mutation. In terms of the expression pattern we found that the expression of CD27 was significantly higher in the case of *BTK* ^C481S^ mutation. (CD27 expression of *BTK* ^C481S^ mutated vs. *BTK* ^C481S^ wild type cases 205.283 vs. 70.692 MFI values, *p* = 0.011). The expression of CD69 did not differ in *BTK* mutant and wild type cases (*p* = 0.176). CD86 showed significantly higher expression in the samples with harbouring *BTK* ^C481S^ mutation than in the wild type samples (*BTK* ^C481S^ mutated vs. *BTK* ^C481S^ wild type 134.28 vs. 33.92 MFI values, *p* < 0.001) (Figure 3).

## Discussion

The novel agents have revolutionized the treatment of CLL in the recent years, however, the emergence of drug resistance has become a severe issue over time. For this reason, there is a huge demand for seeking markers that could predict the treatment response, making it possible to provide personalized therapy. There are numerous molecular techniques which could reliably predict the treatment response during ibrutinib or venetoclax treatment (8,9,24,25,26), while flow cytometry provides only the measurement of MRD and this has been proven to be a reliable technique only in the case of venetoclax treatment (11, 27). According to Ahn et al., unmeasurable MRD level (10^−4^) has been achieved only in few patients’ cases, and there was no significant difference between MRD-high and MRD-low groups in terms of progression free survival among patients treated with ibrutinib (10). Based on this study, MRD level in contrast with the venetoclax treatment did not seem to be a reliable predictor of the treatment response among patients treated with ibrutinib. Hence there still is a need to find novel and reliable flow cytometric markers which could predict the ibrutinib resistance. For this reason, we tried to find such markers in the real-world cohorts of CLL patients. We compared the immunophenotype of treatment naïve (Co *n* = 10), ibrutinib sensitive (IS *n* = 7) and ibrutinib resistant (IR *n* = 11) patient cohorts by using 5 surface markers (CD69, CD184, CD86, CD185, CD27) which are believed to have influence on ibrutinib treatment or disease progression (14,15,16,17,18,19,20).

Concerning CD27, several experiments have been performed in studies, but they have not given unambiguous results. Shen et al. observed that CD27 expression is increased on CLL cells after *in vitro* ibrutinib treatment (18), while Rendeiro et al. found that its expression decreased due to the same treatment (28). Our results tend to confirm Rendeiro’s results. Namely, we found that the expression of CD27 on CLL cells was remarkably lower in the ibrutinib treated cohort than in the treatment naïve samples. Riether et al. suggested that elevated CD27 expression level may play an important role in interplaying between tumour cells and the tumour supporting microenvironment, hence blocking CD27 could be a promising therapeutical approach (29). Gobessi et al. proved that the BCR signalling pathway shows increased activity in Zeta Chain of T Cell Receptor Associated Protein Kinase 70 (ZAP70) positive CLL cells (30). In addition, Lafage et al. observed that the CD27 expression increased in ZAP70^+^ CLL cells (31). Based on these two observations, increased expression of CD27 in CLL cells was accompanied by increased BCR activity, thus it may be possible that the elevated CD27 expression is a sign of the increased activity of the BTK signalling pathway. We compared the CD27 expression of CLL cells in the ibrutinib resistant and the ibrutinib sensitive cohort and found a significantly higher CD27 expression level in the ibrutinib resistant cohort suggesting that CD27 could be a novel biomarker of ibrutinib resistance. It might look promising, but we have not had sufficient results to support this hypothesis yet, therefore further research, including mechanistic studies, are still required.

Del Poeta et al. raised the possibility that CD69 may become a prognostic factor in CLL, because CLL patients with low CD69 expression on CLL cells showed longer progression free survival (32). This hypothesis is supported by the work of Montraveta et al, who proved that the CD69 plays an important role in cell-cell interaction and indicates the bendamustin sensitivity (15). Herman et al. showed that CD69 expression decreased due to ibrutinib (33), and we achieved a similar result. We detected lower level of CD69 expression on CLL cells in ibrutinib sensitive cohort than in treatment naïve cohort. Furthermore, we showed higher level of CD69 expression in the ibrutinib resistant cohort compared to the ibrutinib sensitive cohort. Although, the difference was not significant, but it could be caused by the low number of cases. To sum up, CD69 might be a good candidate for a novel resistance marker of ibrutinib treatment.

According to our results, CD86 also seems to be a promising marker of ibrutinib resistance detected by flow cytometry. Expression of CD86 on CLL cells is significantly lower than on non-tumour B-cells (34), and its expression increased on activated B-cells (35), suggesting that CD86 could be a marker for the activation of CLL cells (36, 37). The expression of CD86 may have a prognostic impact on CLL, because its increased expression is associated with worse prognosis (19, 20). According to Herman et al., both the expression of CD86 and the expression of CD69 are decreased during the ibrutinib treatment (33). In our study the level of CD86 was almost significantly higher in the ibrutinib sensitive group than in the control group (*p* = 0.052). Our results regarding Herman’s work might seem controversial, but it must be taken into account that our control cases contained only treatment naïve patients’ samples while Herman’s cohort was mixed, it had contained treatment naïve and pre-treated patients as well. Therefore, the chemo-immunotherapy might explain the controversy between our results. In addition, in our case-study where the patient was also pre-treated, ibrutinib decreased the expression level of CD86 in the first 3 months of treatment which tend to confirm Herman’s observation. A potential explanation could be that ibrutinib “repairs” the impaired immune functions in CLL (38), and the sign of this phenomenon could be the decrease of the expression level of activation markers. Furthermore, we found that the expression of CD86 on CLL cells was higher in ibrutinib resistant samples compared to ibrutinib sensitive patients’ samples, and this result suggests that the higher CD86 expression could be a sign of ibrutinib resistance. We also examined the presence of BTK^C481S^ mutation among ibrutinib resistant patients and found that the expression of CD86 was significantly higher in *BTK* ^C481S^ mutant samples than in the wild type cases. A plausible explanation could be the following: the CD86 positive CLL cells have higher incidence of DNA damage (20), and this could lead to increased mutational burden, which may explain why *BTK* ^C481S^ mutation is more frequent among CD86 positive CLL cells. Ibrutinib decreases the activation of CLL cells (33), which could be seen as a decrease in the expression of CD86. We have shown that the expression of CD86 was elevated in ibrutinib resistant samples, suggesting that these CLL cells are re-activated by escaping the inhibitory effect of ibrutinib. In conclusion, the elevated expression of the three markers (CD27, CD69, CD86) seem to present the activated state of CLL cells. Although we note the limitation of this observation. The unmutated IgHV status of CLL cells is a well-known marker of the B-cell receptor activation (39, 40) but our cohorts were too small to investigate the correlation between the expression of CD27, CD69 and CD86 and the IgHV mutation status of CLL cells.

We were going to enrol more patients in our prospective study, unfortunately, this study contained only one patient’s data. Therefore, it is barely sufficient to draw a general conclusion. However, we reckon that this study has drawn an attention to a novel issue. Our work revealed that the expression of three surface markers (CD27, CD69 and CD86) was changing similarly during ibrutinib treatment. Their expression level decreased after the beginning of the treatment, then it started to continuously increase, and reached the maximum level at the onset of clinical resistance. Furthermore, these markers’ expression level exceeded their baseline level 3 months prior to the onset of clinical resistance.

## Summary

In our study we have shown that the immunophenotype of CLL cells are different in the samples of ibrutinib sensitive and ibrutinib resistant patients. The expression of CD27 and CD86 was significantly higher in the ibrutinib resistant samples than in ibrutinib sensitive samples. Based on our results, measuring CD27 and CD86 expression by flow cytometry may be a feasible approach besides the detection of *BTK* ^C481S^ mutation to reveal drug resistance during ibrutinib treatment. However, it is still necessary to carry on prospective studies enrolling more patients.

## Data Availability

The datasets used and/or analysed during the current study are available from the corresponding author on reasonable request.
