# Supplementary material for: Revealing a Phenotypical Appearance of Ibrutinib Resistance in Patients With Chronic Lymphocytic Leukaemia by Flow Cytometry
Source: Pathol Oncol Res. 2022 Sep 21;28:1610659. doi: 10.3389/pore.2022.1610659 (PMC9532522; doi:10.3389/pore.2022.1610659)
Supplement: Supplementary file 1 [file DataSheet1.docx]

**Suppl. Table 1** Clinical characteristics of the control patients

| Patient ID | Prior chemo-immunotherapy | Sex | Age at taking sample (year) | IgHV mutation status | TP53 status | CD49d expression at taking sample | CD38 expression at taking sample | BTK^C481S^ mutation status at taking sample |
| --- | --- | --- | --- | --- | --- | --- | --- | --- |
| Co1 | no | F | 74 | NA | del 17p | NP | negative | NP |
| Co2 | no | M | 83 | NP | NP | negative | negative | NP |
| Co3 | no | M | 66 | NP | NP | positive | negative | NP |
| Co4 | no | M | 72 | NP | NP | dim | negative | NP |
| Co5 | no | M | 55 | U | wild type | negative | negative | NP |
| Co6 | no | F | 63 | NP | NP | negative | negative | NP |
| Co7 | no | M | 68 | U | wild type | NP | positive | NP |
| Co8 | no | M | 56 | U | wild type | negative | positive | NP |
| Co9 | no | M | 70 | MU | wild type | NP | negative | NP |
| Co10 | no | F | 80 | NP | NP | negative | negative | NP |

NP: not performed, NA: not avaliable, F: female, M: male, del17p: 17p deletion, U: unmutated, MU: mutated

**Suppl. Table 2** Clinical characteristics of ibrutinib sensitive patients

| Patient ID | Prior chemo-immuno-therapy | Sex | Age at taking sample (year) | IgHV mutation status | TP53 mutation status | Duration of ibrutinib therapy (months) | CD49d expression at 12^th^ month of ibrutinib treatment | CD38 expression at 12^th^ month of ibrutinib treatment | BTK^C481S^ mutation status at 12^th^ month of ibrutinib treatment |
| --- | --- | --- | --- | --- | --- | --- | --- | --- | --- |
| IS1 | yes | F | 86 | NA | NA | 12 | negative | negative | wild type |
| IS2 | yes | F | 76 | NA | NA | 12 | negative | negative | wild type |
| IS3 | yes | M | 63 | NA | NA | 12 | positive | positive | wild type |
| IS4 | yes | M | 63 | U | NA | 12 | negative | positive | wild type |
| IS5 | yes | F | 70 | U | MU | 12 | negative | positive | wild type |
| IS6 | yes | F | 74 | U | MU | 12 | positive | positive | wild type |
| IS7 | yes | F | 72 | NA | MU | 12 | negative | positive | wild type |

NA: not avaliable, F: female, M: male, U: unmutated, MU:mutated

**Suppl. Table 3** Clinical characteristics of ibrutinib resistant patients

| Patient ID | Prior chemo-immuno therapy | Sex | Age at taking sample (year) | IgHV mutation status | TP53 mutation status | Duration of ibrutinib therapy (months) | CD49d expression at presenting the clinical sign of ibrutinib resistance | CD38 expression at presenting the clinical sign of ibrutinib resistance | BTK^C481S^ mutation status at presenting the clinical sign of ibrutinib resistance |
| --- | --- | --- | --- | --- | --- | --- | --- | --- | --- |
| IR1 | yes | M | 77 | U | wild type | 21 | negative | negative | wild type |
| IR2 | yes | M | 71 | U | wild type | 4 | negative | positive | wild type |
| IR3 | yes | F | 74 | NA | wild type | 16 | negative | negative | wild type |
| IR4 | yes | F | 87 | NA. | NA. | 6 | negative | positive | wild type |
| IR5 | yes | M | 62 | B | MU | 25 | positive | positive | MU |
| IR6 | yes | M | 74 | NA | wild type | 32 | negative | positive | MU |
| IR7 | yes | M | 61 | U | MU | 57 | negative | negative | MU |
| IR8 | yes | M | 56 | U | wild type | 36 | negative | positive | MU |
| IR9 | yes | M | 70 | NA. | wild type | 49 | positive | positive | MU |
| IR10 | yes | M | 69 | U | NA | 57 | positive | negative | MU |
| IR11 | NA | M | 70 | U | NA | NA | positive | positive | MU |

NA: not avaliable, F: female, M: male, U: unmutated, B: borderline, MU: mutated

**Suppl. Table 4** Clinical characteristics of the follow-up patient

| Patient ID | Age at starting of venetoclax treatment(years) | Sex | CD49d expression at starting the ibrutinib therapy | CD38 expression at starting the ibrutinib therapy | Cytogenetics (prior to venetoclax treatment) | Duration of venetoclax therapy (days) | Duration of ibrutinib therapy (days) | BTK^C481S^ mutation status at presenting the clinical sign of ibrutinib resistance |
| --- | --- | --- | --- | --- | --- | --- | --- | --- |
| FUP | 76 | M | positive | positive | del13q | 450 | 420 | wild type |

**Suppl. Table 5** Used antibodies and fluorochromes.

| Antigen | Clone | Fluorochrome | Manufacturer |
| --- | --- | --- | --- |
| CD69 | FN50 | FITC | BD Biosciences |
| CD184 | 12G5 | PE | BD Biosciences |
| CD86 | 2331 (FUN-1) | APC | BD Biosciences |
| CD185 | J252D4 | PerCP Cy5.5 | BioLegend |
| CD45 | J33 | PC7 | Beckman Coulter |
| CD19 | J3-119 | AlexaFluor^TM^ 700 | Beckman Coulter |
| CD19 | J3-119 | AlexaFluor^TM^ 750 | Beckman Coulter |
| CD5 | BL1a | ECD | Beckman Coulter |
| CD3 | UCHT1 | APC | Beckman Coulter |
| CD27 | 1A4CD27 | PC5.5 | Beckman Coulter |

Selected markers are surface molecules involved in the pathogenesis and prognosis of CLL

**Suppl. Figure 1** Gating strategy for the determination of CLL cell ratio


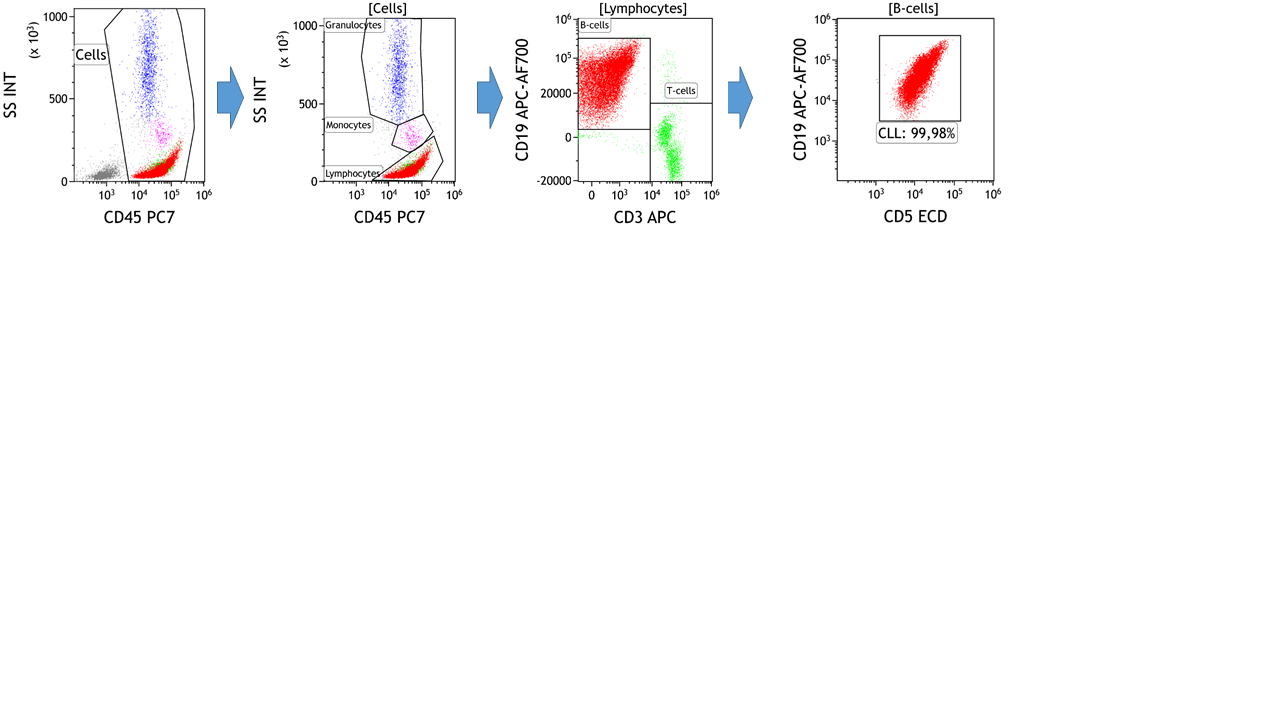


The proportion of CLL cells was assessed by using anti-CD45, anti-CD19, anti-CD5, and anti-CD3 antibodies. Living cells, lymphocytes, monocytes and granulocytes were identified based on side scatter (SSC) and CD45 dot-plots. Finally, the proportion of CLL cells among CD19 positive lymphocytes was assessed by CD5 expression. The CLL cell ratio among B-cells was above 98% in each sample, therefore CLL cells were considered as B-cells.

**Suppl. Figure 2** Gating strategy for the investigated surface markers


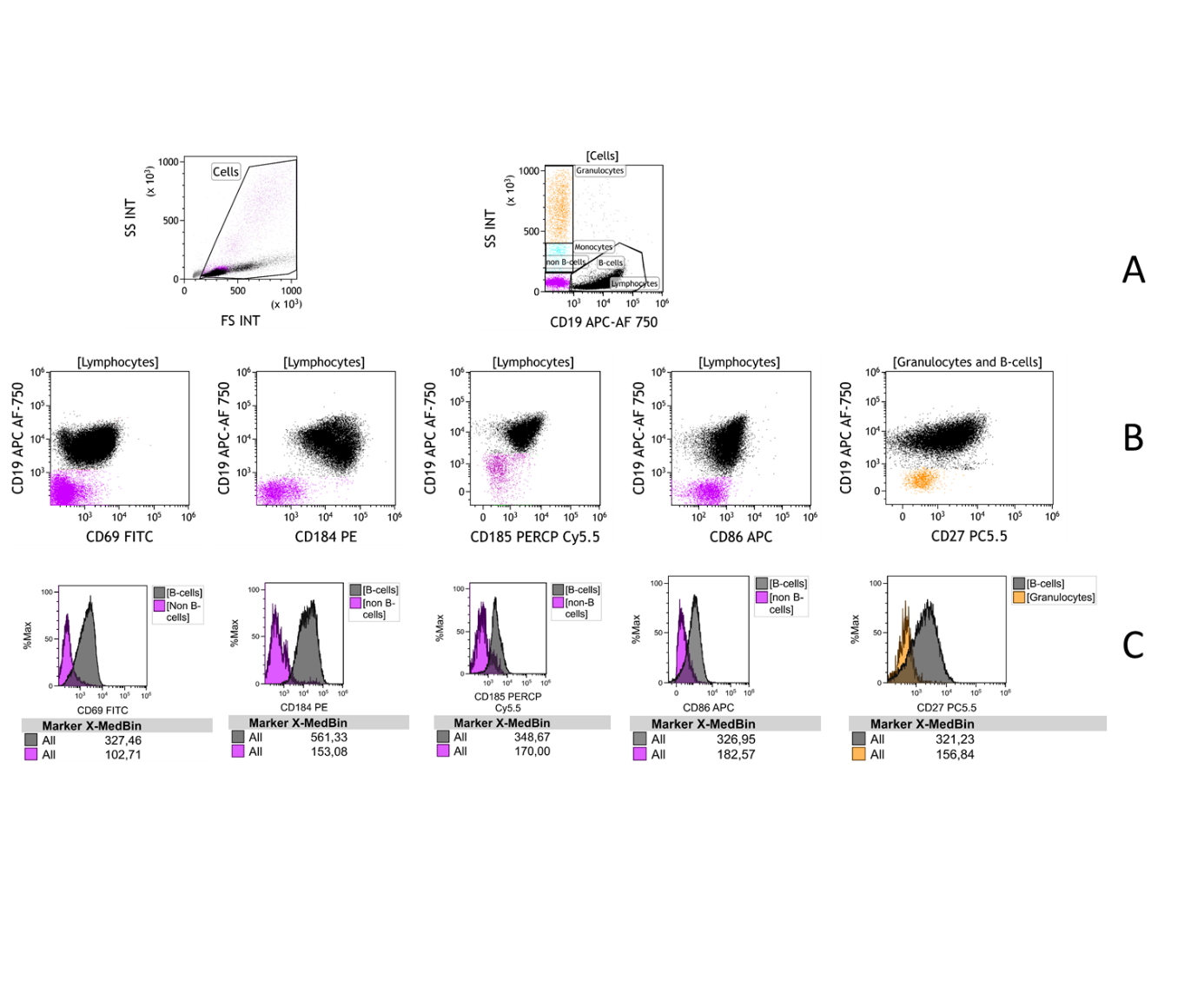


Living cells, lymphocytes, monocytes, granulocytes were identified based on their forward scatter (FSC) and side scatter (SSC) properties, and B-cells by CD19 expression (A). According to our measurements, the CD19 negative lymphocyte population (purple) was suitable as an internal negative control for CD69, CD184, CD185, and CD86. Concerning CD27, the granulocyte population (orange) was used as an internal negative control (B). Calculating the relative MFI value, the MFI value of CD19 negative lymphocytes was subtracted from the MFI value of B-cells (black) to determine the relative expression of CD184, CD185, CD69, and CD86. In case of CD27, the MFI value was calculated by subtracting the MFI value of granulocytes from the MFI value of B-cells (C).

**Suppl. Figure 3** Identification of *BTK^C481S^* resistance mutation by digital droplet PCR.


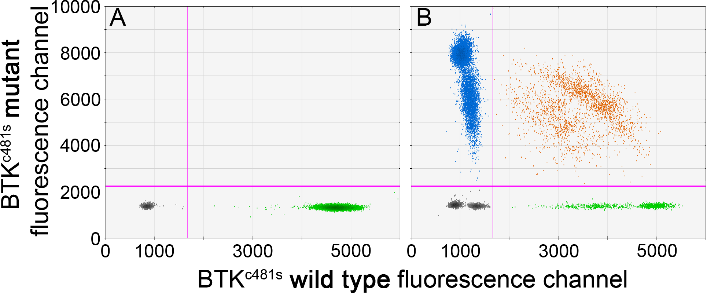


On the representative dot-plots, BTK^C481S^ mutation negative (A) and *BTK^C481S^* positive (B) cases are displayed. The cut-off variant allele frequency (VAF) level was 0%. Samples were considered *BTK^C481S^* positive if *BTK^C481S^* mutation was detected with a VAF higher than 0%. The green dot population represents the droplets containing only wild type DNA. Orange dots represent droplets with wild type and mutant DNA as well, while the blue dot population represents droplets containing mutant DNA only. Grey dots are empty droplets containing water. VAF was calculated in each sample as the ratio of droplets containing mutant DNA (blue) to droplets with wild type DNA molecules (green).
